# Chronic High-Carbohydrate And High-Fat Diet-Induced Metabolic Syndrome Leads To Adrenal Dysfunction, Altered Dendritic Morphology In Limbic Regions, And Anxiety-Like Behaviors In Male Rats

**DOI:** 10.1007/s12031-026-02501-4

**Published:** 2026-03-14

**Authors:** Samuel Treviño, Estefania Fuentes, Berenice Venegas, Guadalupe Muñoz-Arenas, Rubén A. Vázquez-Roque, Fabián Galindo-Ramírez, Everardo Avelino-Cruz, Gonzalo Flores, Jorge Guevara, Alfonso Díaz

**Affiliations:** 1 Institute of Physiology, Benemérita Universidad Autónoma de Puebla, 14 Sur 6301, Puebla, CP 72570 Pue Mexico; 2 Faculty of Medicine, Benemérita Universidad Autónoma de Puebla, Puebla, Pue Mexico; 3 Faculty of Biological Sciences, Benemérita Universidad Autónoma de Puebla, Puebla, Pue Mexico; 4 Faculty of Chemical Sciences, Benemérita Universidad Autónoma de Puebla, Puebla, Pue Mexico; 5 Department of Biochemistry, Faculty of Medicine, National Autonomous University of Mexico, Mexico City, Mexico

**Keywords:** Insulin, Metabolic syndrome, Dendritic order, Caspase-3, Metabolic dysregulation, Dendrites

## Abstract

Metabolic syndrome (MS) represents a major global health problem affecting a large proportion of the population. The improper management of peripheral carbohydrates and lipids leads to behavioral changes in neurons of the limbic brain. This work studied male rats that received diets high in carbohydrates, lipids, and their combination for ninety days. The study examined how peripheral metabolic changes and hypothalamic-pituitary-adrenal (HPA) axis alterations affect dendritic structure in limbic brain regions, thereby inducing anxiety-related behavioral responses. Our results show that male rats develop MS after consuming diets high in fat, carbohydrates, and their mixture for an extended period. These diets after the normal function of the HPA axis, leading to increased anxiety symptoms and reduced dendritic growth in limbic areas. This is accompanied by increased caspase-3 immunoreactivity and decreased cell viability. Therefore, it is suggested that diets high in carbohydrates or fats are a critical factor that could induce adrenal stress that damages dendrites and accelerates neurodegeneration, resulting in anxiety-related behavioral changes.

## Introduction

Metabolic syndrome (MS) represents a complex clinical condition that originates from multiple factors and has become one of the leading causes of chronic metabolic disorders (Neeland et al. 2024, Saklayen 2018). The MS generates various public health issues (Saklayen 2018). Western dietary patterns containing saturated fats and refined foods, induce molecular and cellular changes that affect the central and periphral metabolic control of carbohydrates and lipids (Dhondge et al. 2024, Engin 2017). The body develops peripheral insulin resistance and dysregulation of leptin and cortisol with the consumption of these diets over time. Cortisol triggers insulin production and signaling, which researchers believe leads to the development of insulin resistance.

Research conducted in both laboratory settings and medical facilities shows that the hypothalamic-pituitary-adrenal (HPA) axis regulates body weight (Herman et al. 2016, Sheng et al. 2020). The body consumes more fat- and sugar-rich foods when cortisol levels remain elevated for extended periods, which leads to the development of obesity (Guia et al. 2014, Sapolsky 2021). The body experiences disrupted homeostasis when cortisol levels stay elevated for an extended period. HPA axis dysfunction leads to multiple health problems, including metabolic changes, immune system suppression, and alterations in brain function (Sheng et al. 2020, Radley et al. 2015). The resulting sequence of events creates long-term physiological distress. The mechanism by which it affects both central and peripheral systems remain unidentified, especially in the brain. In vivo, studies in rats and mice show that short- and long-term consumption of high-fat, high-sugar foods increases blood levels of corticosterone, insulin, and glucose (Auvinen et al. 2012, Tsai et al. 2025). In addition to these changes, the animals exhibit stress-related behaviors such as anxiety disorders and memory deficits (Li et al. 2022, Noronha et al. 2019, Treviño et al. 2015). However, there is little evidence that chronic consumption of high-fat and high-sugar diets, in addition to altering energy metabolism, can also alter HPA axis function, which could cause physiological stress and induce anxiety-like behaviors.

Numerous experimental studies using animal models have shown that increased peripheral levels of ACTH and corticosterone are commonly associated with endocrine manifestations of anxiety (Penninx et al. 2021, Shukla et al. 2024). Likewise, it has been suggested that excess corticosterone in the brain is linked to increased neuroinflammation, oxidative stress, and apoptosis, as well as reduced synthesis of neurotrophic factors, leading to brain neurodegeneration in regions such as the hippocampus, frontal cortex, basolateral amygdala (BLA) and hypothalamus (Hassamal 2023).

In our research group, we have found that chronic consumption (3 months) of high-fat and high-sugar diets induces a metabolic alteration similar to MS in humans, accompanied by deficits in spatial and recognition memory in male rats (Fuentes et al. 2023). It also causes oxidative stress, neuroinflammation, reduced dendritic spine density, and neuronal death across limbic regions, including the prefrontal cortex (PFC), hippocampus, and BLA. However, to date, it is not clear whether MS induced by these diets modifies the HPA axis and alters dendritic morphology in limbic regions involved in anxiety processes. Therefore, the present study aimed to evaluate the effects of MS induced by chronic exposure (3 months) to high-carbohydrate and high-fat diets on the responses of physiological stress hormones, dendritic morphology in limbic system neurons of male rats, and their impact on anxiety behaviors.

## Materials and Methods

### Animals

Male Wistar rats of 30 days of age and 100–150 g of body weight (*n* = 48) were provided by the Claude Bernard Bioterium at the Benemerita Universidad Autonoma de Puebla (BUAP). The animals were housed in polycarbonate cages (4 rats per cage) under controlled conditions of temperature and humidity, with a 12:12 h light-dark cycle. They had *ad libitum* access to food and water. The procedures described in this study were approved by the CICUAL-BUAP Ethics Committee (VIEP-2169/2024). They also adhered to the Mexican Official Standard for the Care and Use of Laboratory Animals (NOM-062-ZOO-1999) and implemented the ARRIVE 2.0 guidelines. The research adheres to ethical and regulatory standards by prioritizing animal welfare and minimizing animal distress during experiments. The research team worked to reduce the number of experimental animals per protocol while preserving scientific accuracy, statistical power, and resource efficiency.

### Diets

The research included four experimental groups consisting of twelve rats each that received (1) a standard diet (CD; LabDiet 5001), (2) a High-carbohydrate diet (HCD; Patent: MX/E/2013/047377), (3) a High-fat diet (HFD; LabDiet 5008), and (4) a mixed HCD + HFD diet (50:50). These were provided to the rats for ninety days through unrestricted access to food (Fuentes et al. 2023). The National Academies of Sciences (1996) established minimum nutritional standards for laboratory rats, and all dietary components met these standards. The diet intake in each cage was monitored daily, and the amount consumed was calculated as grams of food consumed multiplied by energy density (kcal/g). The nutrient content in each diet was reported previously (Sánchez-Solís et al. 2021). We documented the complete dietary composition in Table 1. It is important to mention that posterior to the 90 days of diet consumption to generate the MS model, the animals continued to be fed the same diets until the time of euthanasia.

**Table 1:** Estimated nutritional composition of diets. The composition estimated of the CD: Control diet, HCD: High-carbohydrate diet, HFD: High-fat diet and HCD + HFD: mixture of HCD and HFD diets, are shown in the table

	Control Diet (CD) LabDiet^®^5001	HCD Diet MX/E/2013/047377	HFD Diet LabDiet^®^5008	HCD + HFD Diet
Energy	3.67 Kcal/g	4.84 Kcal/g	4.25 Kcal/g	4.25 Kcal/g
Protein	28.507%	7.3%	26.849%	17.08%
Fat	13.496%	5.8%	16.71%	11.26%
Carbohydrates	57.996%	71.4%	56.441%	63.93%
Starch	31.9%	50%	34.9%	42.45%
Glucose	0.22%	10%	0.22%	5.11%
Fructose	0.30%	10%	0.24%	5.12%
Sucrose	3.7%	1.4%	2.57%	1.99%
Lactose	2.01%	0.0%	0.39	0.2%
Fiber	23.566%	0.0%	18.511%	9.255%

### Zoometry

Weekly measurements of body weight and length were taken. A digital scale (Torrey model LPCR-20/40) was used to measure weight, and a tape measure (Lufkin) was used to measure body length from tail base to nose. The calculation of Body Mass Index (BMI) used weight (g) divided by length² (cm), and fat percentage was determined through the Lee index formula: % fat = [(weight in g^^0.33^) / length in mm] × 100.

### Biochemical Assays

The blood samples were collected from anesthetized rats (50 mg/kg sodium pentobarbital) via the tail vein after a 5-hour fast on day 91. Then, the blood sample was centrifuged (400 x g for 10 min); the serum was separated and frozen at -70 ◦C until biochemical analysis.

An oral glucose tolerance test (OGTT) was performed with a 1.75 g/kg anhydrous glucose solution to measure blood glucose levels at 0, 30, 60, and 90 min after the load (Sarmiento-Ortega et al. 2025). Colorimetric assays were performed using a BioSystems BT-330 spectrophotometer to measure baseline glucose and fructosamine. Triglyceride, total cholesterol, low-density lipoprotein (LDL), and high-density lipoprotein (HDL) concentrations were determined using an A15 autoanalyzer (BioSystems, Guadalajara, Mexico) and commercial kits (Spinreact, Spain). Very low-density lipoprotein (VLDL) levels were estimated using the Martin-Hopkins method (Martin et al. 2013). The Statfax 2600 ELISA system from WinerLab Group USA measured serum insulin levels at 0-, 30-, 60-, and 90-minutes (*n* = 12/group) as well as corticosterone and ACTH levels (*n* = 6/group). The homeostatic model assessment of insulin resistance (HOMA-IR) was determined as we previously reported (Potteiger et al. 2002).

### Assessment of Anxiety-Like Behavior

Two days before conducting the anxiety-like behavior tests, the rats in each group were acclimated to the behavior room. Six animals from each group were placed there from 9:00 to 9:45 am. Subsequently, the remaining animals from each group were placed there from 10:00 to 10:45 am. This procedure was performed to minimize the stress of the novel environment that the behavior room might cause.

#### Elevated Cross Maze Test

 The elevated cross maze (ECM) was used as an assessment tool for anxious behavior on day 92 (Moreno-Martínez et al. 2022). The ECM consisted of a 50 cm-high, elevated cross-shaped platform with two open arms 1 cm high and two closed arms with 40 cm-high walls. The behavior room was illuminated by a white light bulb positioned above the maze. A video camera was also mounted directly above the maze on the wall to record the test session for later analysis.

On the day of the test, the animals were placed in the behavior room for 45 min (9:00 am) before the behavioral assessment. To begin the behavioral assessment, six rats per group were placed in the center of the maze, facing a closed arm, and allowed to explore for five minutes. The distance traveled and velocity were recorded on the MEC, as well as the elapsed time and the total number of rat entries into each arm of the maze.

#### Forced Swim Test

The remaining six rats from each group performed the forced swim test on day 92 in the same behavior room (Hernández-Arrambide et al. 2023). A video camera was mounted on a tripod in front of the acrylic cylinder to record the test session for later analysis. On the day of the test, the animals were placed in the behavior room for 45 min (10:00 am) before the behavioral assessment.

This test aims to assess anxiety-like behaviors when coping with stressful conditions. The procedure consisted of two parts: a 15-minute habituation period on day 92 and a 5-minute test on day 93 (with prior acclimatization from 10:00 to 10:45 am). The rats were submerged in an acrylic cylinder filled with water to a depth of 25 cm. The movements of the rats’ limbs were counted per minute. Immobility was defined as the minimum movement necessary to stay afloat without attempting to escape.

### Golgi-Cox Staining and Morphological Analysis

Eight rats from each group were anesthetized before perfusing with 0.9% saline solution on day 94. Brains were extracted and stained using a modified Golgi-Cox method (Flores et al. 2005). The samples were stored in 100 mL amber bottles for thirty days in a dark room at room temperature. Subsequently, the brains were transferred to a 30% sucrose solution in 100 mL amber bottles at room temperature for 3 days, and the bottles were kept in a dark room.

A vibratome (LEICA VT 1000 S) was used to cut brains into 200-µm coronal sections. Tissue sections were placed on clean gelatin-coated slides and treated with ammonium hydroxide for 30 min, followed by 30 min in Kodak film fixer. Finally, they were rinsed with distilled water and mounted in a resin medium. The researchers studied neurons from PFC layers III and V, the CA1, CA3, and DG regions of the hippocampus, the BLA, and the NAcc.

A trained observer, unaware of the experimental conditions, traced the neurons on both sides using a camera lucida at 250X magnification (DMLS, Leica Microscope). Sequential two-dimensional reconstructions of the entire dendritic tree of each neuron were generated, and Sholl’s analysis quantified the dendritic traces (Sholl 1953) as follows: a transparent grid with equidistant concentric rings (10-µm) was centered over the dendritic tree traces, and the number of ring intersections was used to estimate the total dendritic length and dendritic arborization (Flores et al. 2005). The total number of dendritic branches (a forked Y indicates branching), another estimate of dendritic arborization, was counted at each order of magnitude of distance from the soma or dendritic axis. The researchers reconstructed and analyzed 10 neurons for each brain region of interest; in total, 560 neurons were analyzed per experimental group.

### Caspase-3 Immunohistochemistry

Four rats per group were anesthetized with sodium pentobarbital (50 mg/kg) and perfused with 4% paraformaldehyde. Brains were post-fixed, paraffin-embedded, and coronally sectioned (5-µm). Sections were deparaffinized, rehydrated, and blocked with 2% BSA. Tissue was permeabilized with 0.2% Triton X-100 and incubated with primary anti-caspase-3 antibody (1:100, Santa Cruz Biotechnology; Cat# sc-7272, RRID: AB_626803) followed by rhodamine-labeled secondary antibody (1:100, Jackson Immuno Research Laboratories Inc., Cat# 111-095-144, RRID: AB_2337978). Nuclei were counterstained with DAPI (VectaShield, Vector Labs; CA Cat: H-1000, RRID: AB_2336789). Fluorescence was visualized using an Olympus BX-41 microscope. Images were analyzed using Image-Pro Premier software. Fourteen fields per region were quantified by a morphology expert in a blinded manner. Tissues were observed at 40X magnification. The number of Caspase-3-immunoreactive cells in the PFC, CA1, CA3, DG, BLA, and NAcc was counted over a 500-mm^2^ area. Fourteen fields (semiquantitative analysis) per slide were analyzed and graphically presented as the mean ± standard deviation per group.

### Cell Viability Assay

Viability was determined by acridine orange (AO) staining. A working solution was prepared by diluting 2 mg/mL of AO in distilled water (1:100) (Moroni-González et al. 2024). Sections were rehydrated, washed, and placed in the AO solution for 5 min at room temperature. Finally, the tissues were washed with PBS, dried, and 50-µL of Vectashield was added, and they were sealed. AO interacts with DNA and RNA through intercalation or electrostatic attraction, respectively. DNA intercalated with AO fluoresces green (viable cells); RNA or denatured DNA has electrostatically bound AO, fluorescing red (apoptotic cells). Viability was determined using a fluorescence microscope equipped with a camera (Olympus BX-41). Cell counting was performed using ImageJ version 1.53, included with 64-bit Java 8 for Windows, on separate channels. Cell counts were expressed as a percentage of viability in each brain region of interest over a 500-mm^2^ area.

### Statistical Analysis

Data are expressed as mean ± standard deviation (SD). Data normality was determined by the Shapiro-Wilk test. Zoometry, metabolic parameters, anxiety behaviors (Distance traveled, velocity, time spent in open and closed arms and number of limb movements), corticosterone, ACTH, and total dendritic length (TDL) were analyzed by one-way ANOVA. Results were reported as an F-statistic—the ratio of the variance between groups to the variance within groups—and the analysis was completed with a Bonferroni post hoc test when F < 2.95 (F-critical), with *p* < 0.05 considered significant. Meanwhile, branching order length was analyzed using a two-way ANOVA. The statistical interaction (F) corresponds to the mean dendrite length of a specific brain region in relation to dendritic tree morphology, the number of dendritic intersections, and dietary treatment. Their interaction was assessed using the F-statistic, calculated as the time factor mean square divided by the residual mean square, with degrees of freedom used to determine the F-statistic distribution. The Kruskal-Wallis test was used to compare the semiquantitative results for Caspase-3 and cell viability. Data analysis was performed with GraphPad Prism 10 (GraphPad Software Inc., USA). (*) A significant level of *p* ≤ 0.05 was considered in comparison to the respective control group.

## Results

### Diets High in Carbohydrates, Fats, and their Combination Modify Zoometric Parameters

After 90 days of feeding the animals with HCD, HFD, and HCD + HFD diets, the zoometric parameters were altered with respect to CD (Table 2). Rats fed with HCD and HCD + HFD showed a significant increase in body weight (10.7% and 23%, *p* < 0.01; F = 3.169), BMI (12.5% and 23%, *p* < 0.01; F = 5.785), and as we expected, all experimental groups showed a significant increase in the Lee index: HCD (30%), HFD (28%), and HCD + HFD (35%) (*p* < 0.01; F = 7.913). These results indicate increased adiposity, which is associated with MS.

**Table 2:** Diets high in carbohydrates, fats, and their mixture modify the zoometric parameters in male rats. The results shown are the average of zoometric parameters (*n* = 12/group) ± SD. (**) *p* < 0.01 indicates significant difference compared to control group by One-way ANOVA test followed Bonferroni post hoc test. BMI: Body Mass Index

Zoometric parameters	CD Lab Diet 5001 (*n* = 12)	HCD MX/E/2013/047377 (*n* = 12)	HFD Lab Diet 5008 (*n* = 12)	HCD + HFD (*n* = 12)
Body Weight (g)	342.7 ± 9.37	379.6 ± 6.37**	350.6 ± 11.35	420.8 ± 10.96**
Height (cm)	20.65 ± 1.23	20.48 ± 0.72	20.78 ± 0.57	20.55 ± 0.39
BMI (g/cm^2^)	0.80 ± 0.08	0.90 ± 0.04**	0.81 ± 0.05	0.99 ± 0.04**
Body Fat (%) Lee index	3.29 ± 0.41	4.28 ± 0.25**	4.23 ± 0.22**	4.45 ± 0.34**

### Diets High in Carbohydrates, Fats, and their Mixture Disrupt Carbohydrate and Lipid Homeostasis

**Fig. 1:**
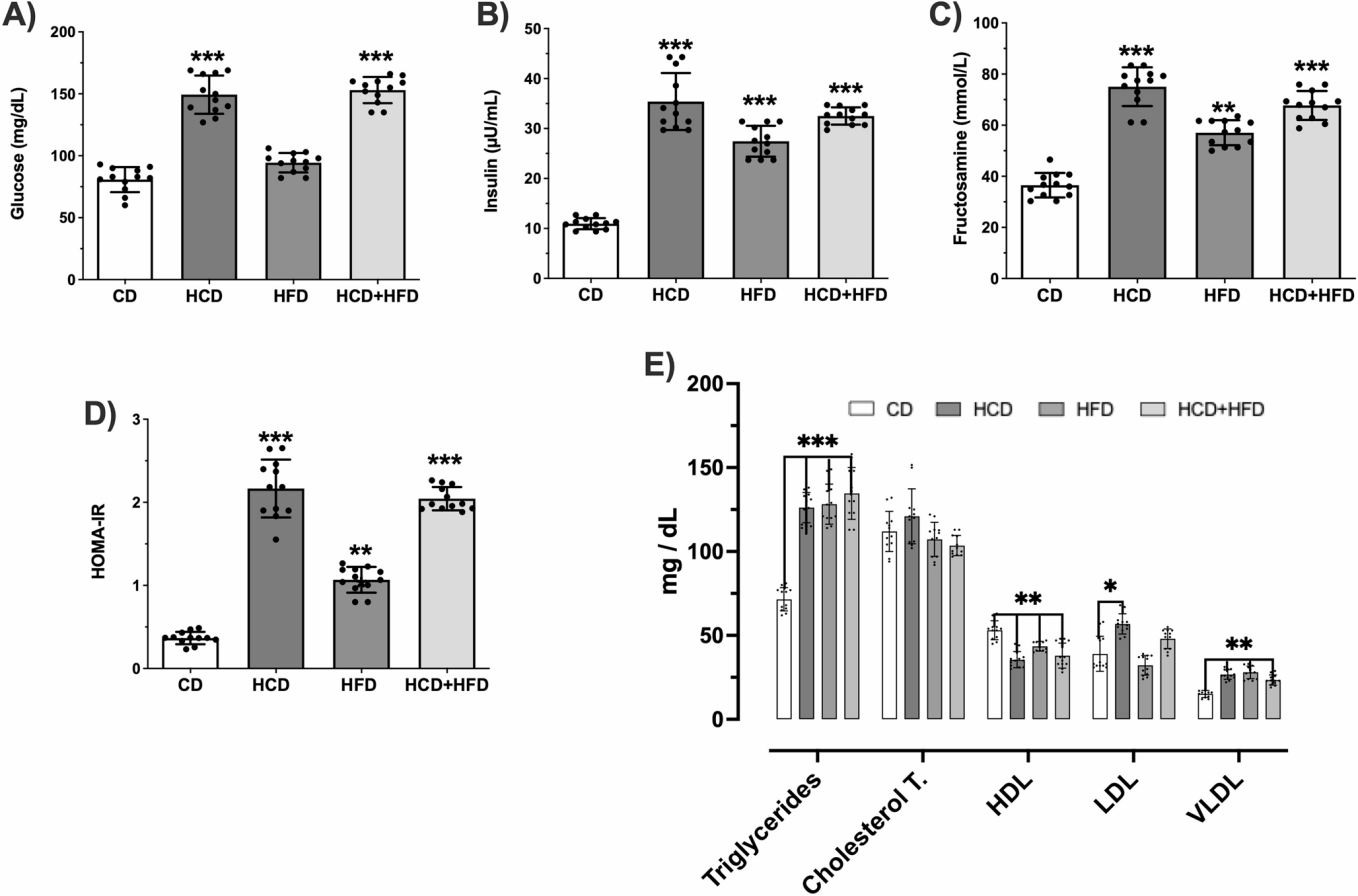
Effect of high- diet consumption on serum carbohydrate and lipid metabolism in male rats. After treatment, the animals in each experimental group (*n* = 12) had their blood levels determined: Glucose (**A**), Insulin (**B**), Fructosamine (**C**), HOMA-IR, Triglycerides, Total Cholesterol, HDL, LDL and VLDL (**D**). The mean of data ± SD is plotted. Data were analyzed with One-way ANOVA followed Bonferroni post hoc test *p < 0.05, ∗∗*p* < 0.01, and ∗∗∗*p* < 0.001 compared with the CD group)

The biochemical results of MS hallmarks were compared between HCD, HFD, the mixture group, and the control group (CD) (Fig. 1). Fasting glucose levels (Fig. 1A) in the HCD and HCD + HFD groups increased by 84% and 90% (*p* < 0.001; F = 129.3). Fasting insulin levels (Fig. 1B) increased significantly in the experimental groups: 223% in HCD, 151% in HFD, and 197% in HCD + HFD (*p* < 0.001; F = 123.7). Fructosamine levels (Fig. 1C) were significantly elevated in all groups: 105% in HCD, 56% in HFD, and 85% in HCD + HFD (*p* < 0.001; F = 98.86). Besides, the HOMA-IR index also increased significantly: 513% in HCD, 146% in HFD, and 486% in HCD + HFD (*p* < 0.001; F = 204.4) (Fig. 1D). Clearly indicating a loss of homeostasis in the metabolism of carbohydrates at the serum level due to the consumption of these diets.

The lipid profile showed metabolic dyslipidemia (Fig. 1E). Hypertriglyceridemia was observed in all groups by 76% in HCD, 79% in HFD, and 88% in HCD + HFD (*p* < 0.0001; F = 36.44). Total cholesterol levels did not differ significantly between groups (*p* = 0.1095; F = 2.289). However, the cholesterol fractions exhibited quantitative changes. VLDL cholesterol increased by 76% in HCD, 83% in HFD, and 56% in HCD + HFD (*p* < 0.0001; F = 20.86). LDL cholesterol was significantly elevated only in the HCD group (45%; *p* < 0.05; F = 13.22). HDL cholesterol levels decreased significantly by 33% in HCD, 21% in HFD, and 28% in HCD + HFD (*p* < 0.01; F = 11.39). These results show evidence that chronic consumption of any of these diets causes dyslipidemia.

**Fig. 2:**
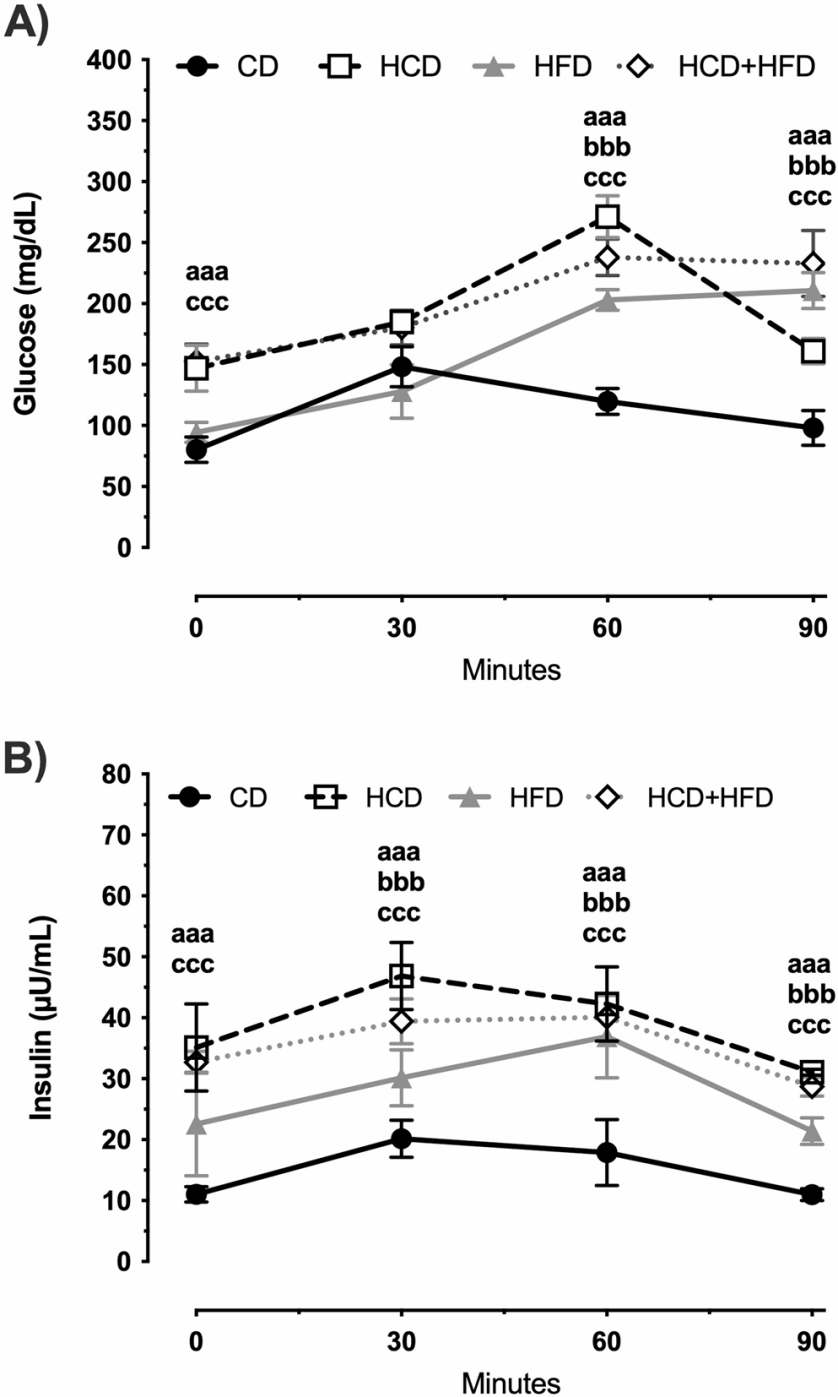
Diets rich in carbohydrates, fats, and their mixture cause peripheral insulin resistance in male rats. The glucose tolerance test and insulin response were performed in conscious animals (*n* = 12/group). A glucose load (1.75 g/kg) was administered orally, and blood glucose and insulin levels were measured at 0, 30, 60, and 90 min after the load. The mean of data ± SD is plotted. Data were analyzed with One-way ANOVA followed Bonferroni post hoc test; ^aaa^*p* < 0.001 to compare the HCD group and CD; ^bbb^*p* < 0.001 to compare the HFD group and CD and ^ccc^*p* < 0.001 to compare the HFD group and CD

Oral glucose tolerance tests and corresponding insulin responses were performed for all groups (Fig. 2). At time 0, glucose levels were significantly higher in the HCD and HCD + HFD (*p* < 0.001; F = 129.3) groups compared to the CD group. At 30 min post-glucose loading, no significant differences were observed between the experimental groups (*p* = 0.0137; F = 4.620). However, at 60 and 90 min, glucose levels were significantly higher in all nutrient-rich diet groups: HCD (126% and 64%), HFD (69% and 114%), and HCD + HFD (98% and 138%) (*p* < 0.0001; F = 193.6 and *p* < 0.0001; F = 87.96).

The insulin response to the glucose load also showed a significant increase at fasting (0 min) in the HCD (217%) and HCD + HFD (197%) (*p* < 0.001; F = 123.7) groups relative to the control. At 30, 60, and 90 min, insulin levels significantly increased in the HCD group (132%, 137%, and 184%), in the HFD group (49%, 107%, and 99%), and in the HCD + HFD group (96%, 124%, and 163%) (*p* < 0.0001; F = 51.15, *p* < 0.0001; F = 31.22 and *p* < 0.0001; F = 290). Collectively, our results indicate that exposure to diets high in sugars, fats, and their mixture dysregulates the regulation of carbohydrate and lipid metabolism.

### Diets High in Carbohydrates, Fats, and their Mixture Increase Corticosterone and ACTH in the Blood

**Fig. 3:**
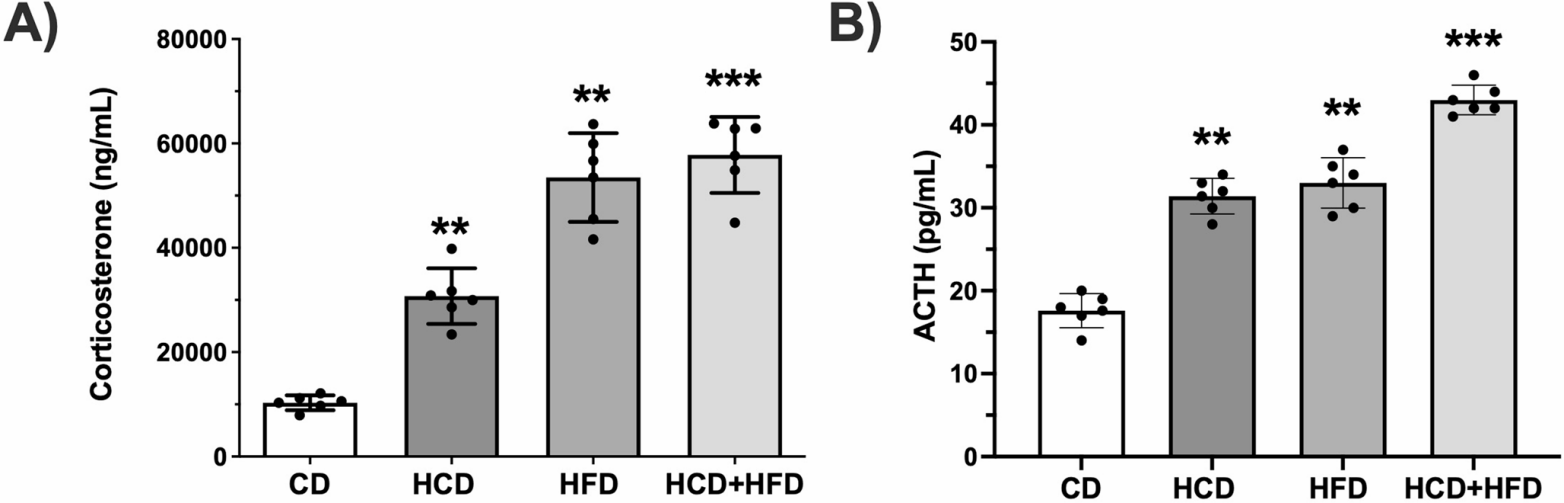
Diets high in carbohydrates, fats, and their mixture increase hypothalamic-pituitary-adrenal (HPA) axis activity in the blood of male rats. After treatment, the concentration of corticosterone (**A**) and ACTH (**B**) in the blood of each experimental group chronically exposed to diets high in carbohydrates, fats and their mixture was quantified in the animals of each experimental group (*n* = 6). The mean of data ± SD is plotted. Data were analyzed with One-way ANOVA followed Bonferroni post hoc test (**p < 0.01 and ***p < 0.001, compared with the CD group)

The study evaluated peripheral hormonal stress factors by measuring corticosterone, and ACTH levels in serum (Fig. 3A-C). The corticosterone and ACTH levels were elevated in the HCD group (198%, and 78%), in HFD (419%, and 87%), and in the HCD + HFD (461%, and 144%) (*p* < 0.0001; F = 74.70 and *p* < 0.0001; F = 123.1). These findings clearly demonstrate that the HCD, HFD, and HCD + HFD diets are an important factor in raising stress hormones, and the effect is most pronounced when animals receive the combined HCD + HFD diet.

### Chronic Consumption of Diets Rich in Carbohydrates, Fats, and their Mixture Induces Anxiety

**Fig. 4:**
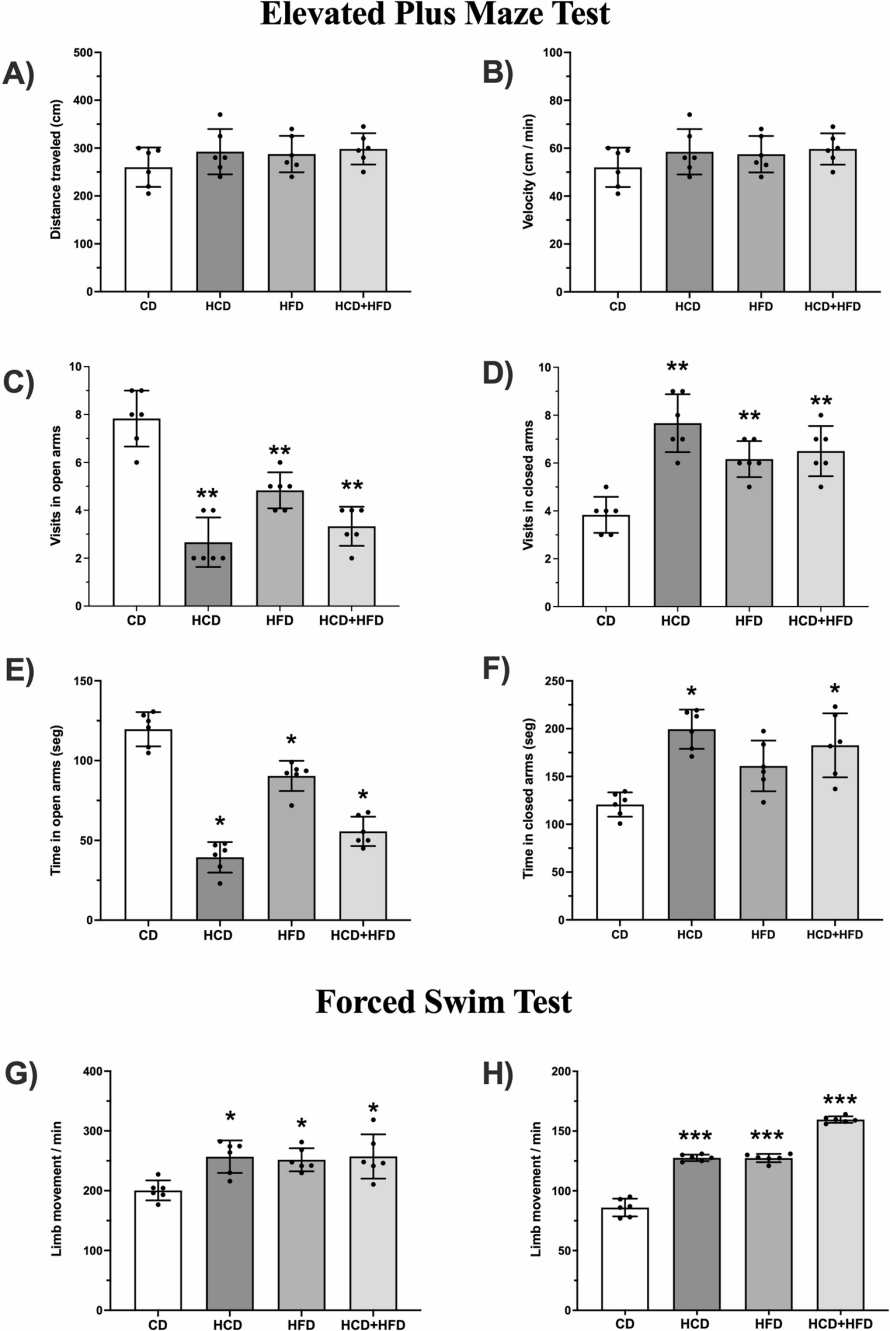
Chronic consumption of diets high in carbohydrates, fats, and their mixture increases anxiety-like behavior in male rats. To assess anxiety-like behaviors, experimental animals were exposed to the elevated cross maze. (*n* = 6) and performed the forced swim test (*n* = 6). In the elevated cross-maze task (**A**-**D**), the number of visits and the time spent in seconds in both the open and closed arms were quantified, respectively. In the forced swim test (**E**), the number of limb movements per minute was quantified for each of the animals treated with diets high in carbohydrates, fats and a mixture of them. The mean of data ± SD is plotted. Data were analyzed with One-way ANOVA followed Bonferroni post hoc test (∗*p* < 0. 05; ∗∗*p* < 0.01, and ∗∗∗*p* < 0.001 compared with the CD group)

Anxiety levels were evaluated under two different conditions: the ECM test to assess anxiety under non-stressful conditions, and the forced swim test to assess anxiety under stressful conditions (Fig. 4).

In the ECM test, initially, the distance traveled (Fig. 4A) and velocity (Fig. 4B) of each group of animals were quantified. The results indicate that diets high in carbohydrates, fats, and their combination, compared to the group fed with CD, did not modify the distance traveled (CD: 200.4 ± 16.67 cm; HCD: 256.9 ± 27.25 cm; HFD: 251.7 ± 19.28 cm; HCD + HFD: 257.2 ± 37.05 cm) (*p* = 0.3826; F = 1.074) or velocity (CD: 52 ± 8.22 cm/min; HCD: 58.5 ± 9.46 cm/min; HFD: 57.50 ± 7.60 cm/min; HCD + HFD: 59.67 ± 6.53 cm/min) (*p* = 0.3826; F = 1.074) during their stay in the ECM. This suggests that feeding with the different diets studied did not alter motor activity.

Furthermore, animals fed diets rich in carbohydrates, fats, and their combination showed a reduced number of visits with open arms (Fig. 4C) compared to the control group (CD: 7.8 ± 1.16; HCD: 2.6 ± 1.03; HFD: 4.8 ± 0.75; HCD + HFD: 3.3 ± 0.81) (*p* < 0.0001; F = 34.55). In contrast, the CD group made fewer visits into the closed arms (CD: 3.83 ± 0.75) than the experimental groups (HCD: 7.6 ± 1.2; HFD: 6.1 ± 0.75; HCD + HFD: 6.5 ± 1.04) (*p* < 0.0001; F = 16.74) (Fig. 4D).

The time spent in the open arms (Fig. 4E) was significantly lower in the groups with diets rich in carbohydrates, fats, and their mixture (HCD: 39.4 ± 9.5 s; HFD: 90.4 ± 9.4 s; HCD + HFD: 55.6 ± 9.2 s) than in the control group (CD: 119.6 ± 10.7 s) (*p* < 0.05; F = 81.38). While the time spent in the closed arm (Fig. 4F) was only significant in animals fed with HCD (199.4 ± 20.5s) and HCD + HFD (182.6 ± 33.4 s) compared to the control group (CD:120.6 ± 12.7 s) (*p* < 0.05; F = 11.55). Statistical analysis of the data suggests that animals fed CD tend to develop non-anxious behavior compared to animals treated with high-nutrient diets under non-stressful conditions.

In the forced swim test (Fig. 4G), during the habituation phase, animals fed HCD, HFD, and HCD + HFD showed a significant increase of 28%, 27%, and 29% in limb movements per minute compared to the group fed CD (*p* < 0.05; F = 6.582). This behavior was interestingly replicated 24 h after the habituation phase. The data reveal that the number of upper and lower limb movements increased by 48% in the HCD group, 48% in the HFD group, and 85% in the HCD + HFD group (*p* < 0.001; F = 265.4) (Fig. 4H), respectively. The results indicate that animals under stressful conditions and with chronic consumption of high-nutrient diets exhibit a greater number of limb movements in both the habituation and final evaluation phases. Consequently, it is associated that a stressor makes anxious behavior evident in the animals. The results using both models suggest that animals consuming nutrient-rich foods are more likely to develop anxiety-like behaviors, so a diet high in carbohydrates, fats, and their combination could be considered one of the multiple factors that cause anxiety.

### Chronic Consumption of Diets High in Carbohydrates, Fats, and Mixed Diets Induces Neuronal Hypotrophy

To evaluate the impact of MS induced by diets rich in carbohydrates, fats, and the mixture on neuronal structure, we performed Golgi-Cox staining and analysis of total dendritic length (TDL) and branching order of pyramidal neurons in the PFC (layers III and V), the CA1 and CA3 regions of the hippocampus, granule cells in the dentate gyrus (DG) and basolateral amygdala (BLA), as well as the medium spiny neurons in the nucleus accumbens (NAcc).

**Fig. 5:**
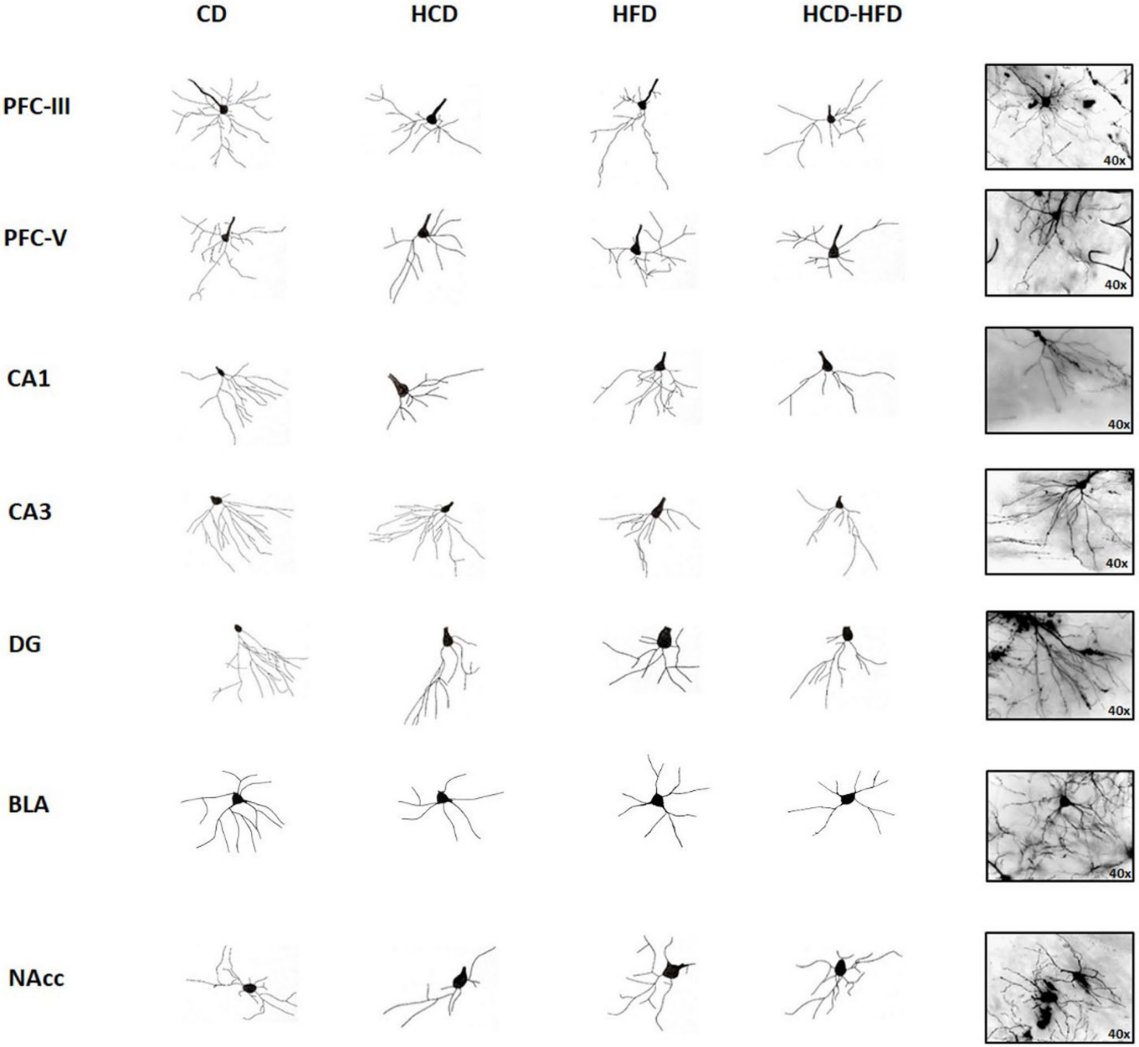
Diagram of the dendritic tree of limbic system neurons in male rats fed diets high in carbohydrates, fats and their mixture for 90 days. The panel shows representative neurons of the neurons of interest and a schematic of the dendritic arbors of neurons in the prefrontal cortex (PFC) layers 3 and 5; hippocampus CA1 and CA3, as well as the dentate gyrus (DG), basolateral amygdala (BLA) and nucleus accumbens (NAcc)

**Fig. 6:**
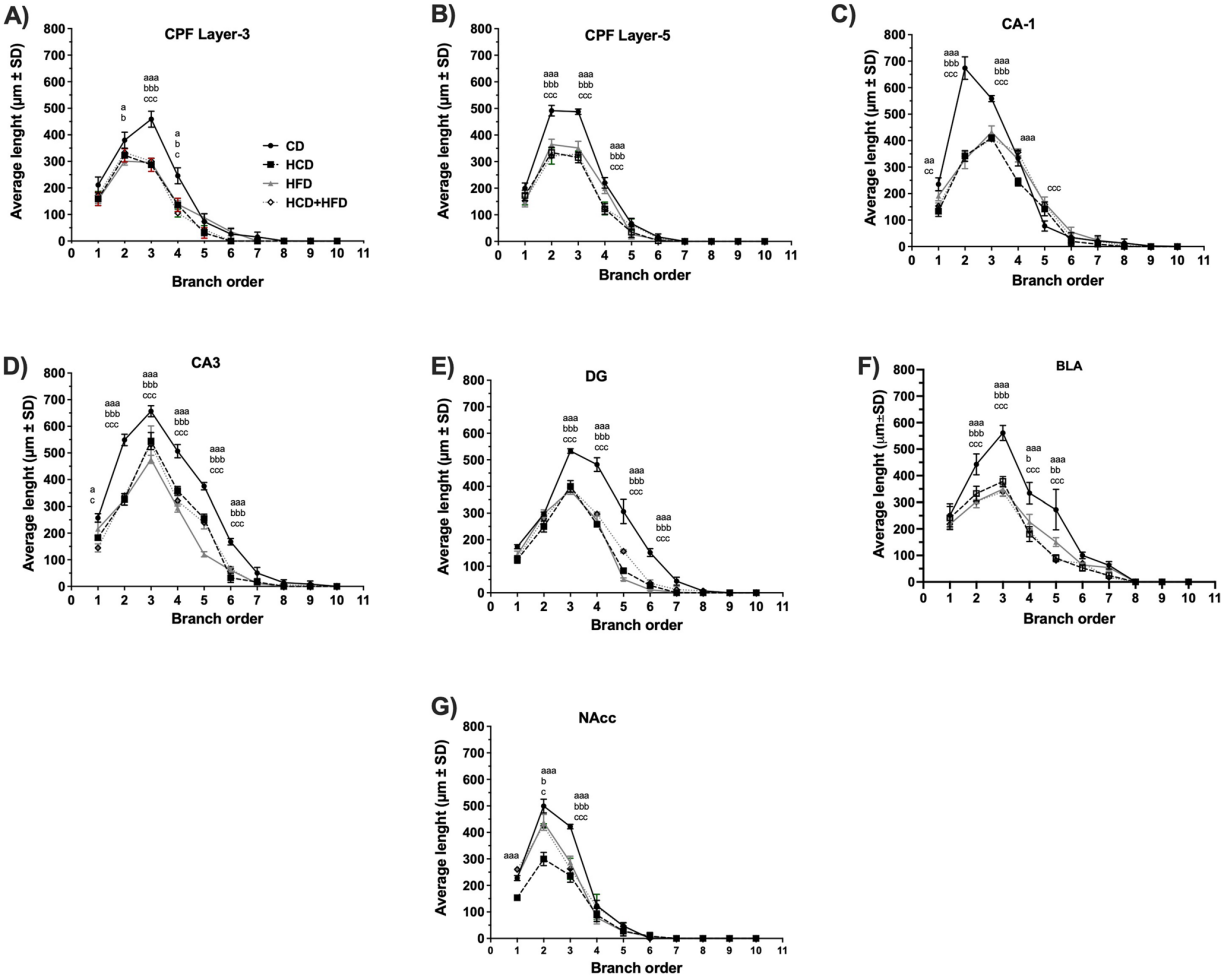
Effects of diets high in carbohydrates, fats, and their mixture on dendritic length according to branching order in limbic brain regions of male rats. Abbreviations: (PFC) layers 3(**A**) and 5(**B**); hippocampus CA1(**C**) and CA3(**D**), as well as the dentate gyrus (DG)(**E**), basolateral amygdala (BLA) (**F**) and nucleus accumbens (NAcc)(**G**). The Average Length (µm) ± SD (*n* = 8/ group) is plotted. Data were analyzed with Two-way ANOVA; ^a^*p* < 0.05, ^aa^*p* < 0.01 and ^aaa^*p* < 0.001 to compare the HCD group and CD; ^b^*p* < 0.05, ^bb^*p* < 0.01 and ^bbb^*p* < 0.001 to compare the HFD group and CD and ^c^*p* < 0.05, ^ccc^*p* < 0.01 and ^ccc^*p* < 0.001 to compare the HCD + HFD group and CD

**Fig. 7:**
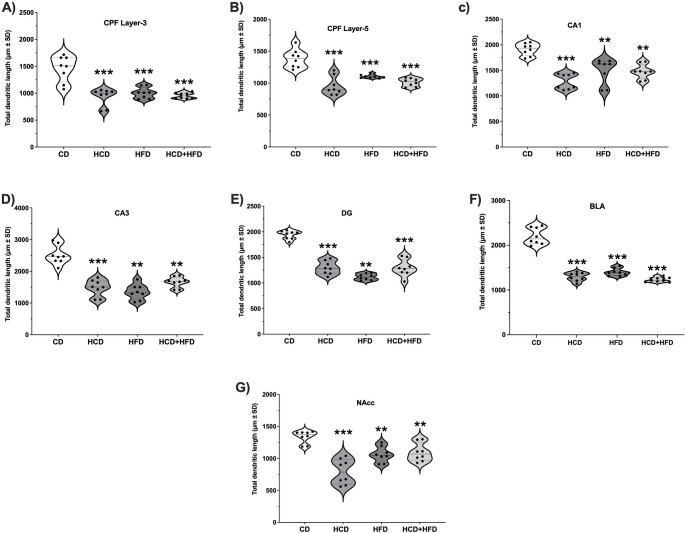
Evaluation of total dendritic length (TDL) in limbic regions of male rats fed diets high in carbohydrates, fats, and their mixture for 90 days. Abbreviations: (PFC) layers III (**A**) and layer V (**B**); hippocampus CA1(**C**) and CA3(**D**), as well as the dentate gyrus (DG)(**E**), basolateral amygdala (BLA) (**F**) and nucleus accumbens (NAcc)(**G**). The dots represent the average LDT of neurons per rat ± SD (*n* = 8/group). Data were analyzed with One-way ANOVA followed Bonferroni post hoc test ∗∗*p* < 0 01 and ∗∗∗*p* < 0 001 compared with the CD group

Figure 5 presents representative photomicrographs of neurons from the brain regions of interest after 3 months of diet consumption. Sholl analysis to determine the branching order of pyramidal neurons of layer III pyramidal neurons in the PFC (Fig. 6A) revealed a high interaction between HCD, HFD, HCD-HFD consumption, and reduction in dendritic arborization from orders 2 to 4 in the HCD and HFD groups (F3,28 = 77.83, *p* < 0.001). In contrast, orders 3 and 4 in the HCD + HFD group (F3,28 = 4.88, *p* = 0.0077) showed a lower dependency on high-nutrient intake, despite diminished dendritic arborization. In layer V (Fig. 6B), the HCD group showed a reduction in orders 2 to 4 (F3,28 = 104.88, *p* = 0.0001), the HCD + HFD group from orders 2 to 4 (F3,28 = 71.23, *p* = 0.0001), and the HFD group from orders 2 to 4 (F3,28 = 78.81, *p* < 0.0001). Layer V showed a high susceptibility to metabolic conditions induced by a diet high in carbohydrates, fats, or their combination. The TDL in pyramidal neurons of layers III and V of the prefrontal cortex (PFC) of the experimental groups is shown in Fig. 7A and B. In the graphs, the small black circles represent the average TDL of each animal per group. Data analysis indicates that animals exhibit lower TDL in the HCD (36% and 31%), HFD (31% and 20%), and HCD + HFD (34% and 26%) groups (*p* < 0.001; F = 20.26, in layer III and *p* < 0.001; F = 25.16, in layer V) compared to the CD group.

In the hippocampus, pyramidal neurons in the CA1 region (Fig. 6C) showed reduced dendritic complexity in the HCD group (orders 1–4; F3,28 = 140.5, *p* < 0.001) and in the HCD + HFD group (orders 1–3 and 5; F3,28 = 180.2, *p* < 0.001), while the HFD group reduced at orders 2 and 3 (F3,8 = 2.25, *p* < 0.001). In the CA3 region (Fig. 6D), dendritic arborization was significantly reduced at from orders 1 to 6 in the HCD and HCD + HFD groups (F3,28 = 66.24, *p* < 0.001), and at orders 2 to 6 in the HFD group (F3,28 = 150.1, *p* < 0.001). Similarly, in DG granule neurons (Fig. 6E), all groups that followed a diet high in carbohydrates, fats, and their mixture showed significant reductions in dendritic branching at orders 3 to 6 compared to controls (F3,28 = 53.02, *p* < 0.0001; F3,28 = 27.91, *p* = 0.0006; F3,28 = 50.81, *p* < 0.0001).

Similarly, when evaluating the average LDT of the animals in each experimental group, Fig. 7C and D show that, on average, the hippocampal neurons in the CA1, CA3, and DG regions of the animals fed high-nutrient diets decreased significantly (HCD: 32.7, 41.0, and 33.1%), (HFD: 22.1, 46.3, and 42.3%), and (HCD + HFD: 22.7, 33.8, and 33.2%)(CA1; *p* < 0.001; F = 18.85),(CA3; *p* < 0.001; F = 36.46), (DG; *p* < 0.001; F = 77.08). This demonstrates that chronic consumption of foods high in carbohydrates, fats, and their combination causes dendritic hypotrophy in the CA1, CA3 and DG of the animals, clearly contrasting with the neurons of the animals that consumed a standard diet (CD).

In the pyramidal neurons, the basolateral amygdala (BLA) showed decreased arborization complexity (Fig. 6F). The HCD, HFD, and HCD + HFD groups revealed significant reductions at dendritic orders 2 to 5 (F3,28 = 138.32, *p* < 0.0001). When analyzing the average TDL of the rats that make up each experimental group, the data confirm that there is a significant reduction in TDL of the animals in the HCD group (41.2%), HFD (36.5%) and HCD + HFD (44.2%) compared to the CD group (Fig. 7F) (*p* < 0.001; F = 123.6).

Finally, in the nucleus accumbens (NAcc), medium spiny neurons exhibited decreased arborization complexity (Fig. 6G). The HCD group showed significant reductions at dendritic orders 1 to 3 (F3,28 = 83.74, *p* < 0.0001), the HFD group at orders 2 and 3 (F3,28 = 31.23, *p* < 0.0001), and the HCD + HFD group at orders 2 and 3 (F3,28 = 51.74, *p* < 0.0001). While the average dendritic length (DL) in neurons of animals fed HCD, HFD, and HCD + HFD was significantly reduced (40.7%, 20.6%, and 18.1%) compared to the CD group (*p* < 0.001; F = 19.40) (Fig. 7G). Morphological analysis suggests that chronic consumption of high-nutrient diets is a critical factor in causing a loss of arborization and dendritic length in limbic system neurons in male rats.

### Diets High in Carbohydrates, Fats, and their Mixture Modify Caspase-3 Immunoreactivity and Cell Viability

**Fig. 8:**
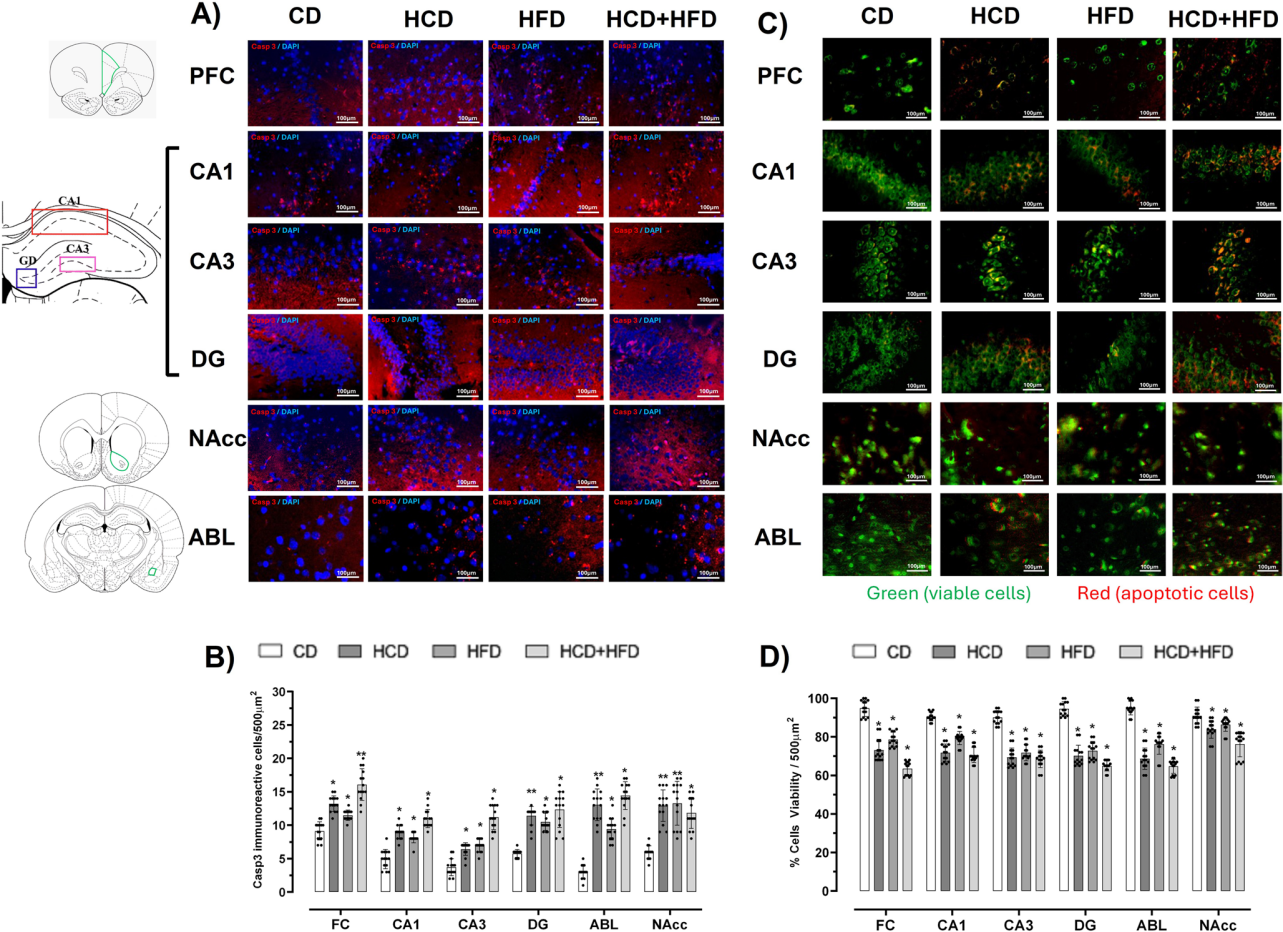
Diets rich in carbohydrates, fats, and their mixtures increase caspase-3 (Casp-3) immunoreactivity and decrease cell viability in the limbic system of male rats. In (**A**) and (**C**) Representative photomicrographs are shown that demonstrate immunoreactivity for casp-3 (red color) and acridine orange (green color: viable cells and red color: apoptotic cell) in the prefrontal cortex (PFC); hippocampus CA1 and CA3, as well as in the dentate gyrus (DG), basolateral amygdala (BLA) and nucleus accumbens (NAcc). (**B**) and (**D**) show the number of Casp-3 immunoreactive cells/ 500 µm^2^ and % cell viability/500 µm^2^ in the study regions of interest. The mean of data ± SD (*n* = 4/ group) is plotted. Data were analyzed with Kruskal-Wallis test (∗*p* < 0 05 and ∗∗*p* < 0 01, compared with the CD group)

Caspase-3 (Casp-3) immunoreactivity was analyzed in brain regions of interest. Qualitative analysis (Fig. 8A) revealed increased immunoreactivity in all areas examined in the three experimental groups, with the most pronounced changes observed in animals exposed to the HCD. Semi-quantitative analysis (Fig. 8B) further supported these findings. By showing that in the PFC (*p* < 0.001; F = 50.10), CA1 (*p* < 0.001; F = 73.58), CA3 (*p* < 0.001; F = 82.28), DG (*p* < 0.001; F = 38.75), BLA (*p* < 0.001; F = 100.1)and NAcc (*p* < 0.001; F = 44.0) there was greater immunoreactivity to Cap-3 in the groups with HCD (44.2, 83.6, 72.9, 100, 93.2 and 118.6%,), HFD (12.1, 66.1, 86.4, 84.2, 94.3 and 123.7%) and HCD + HFD (75.8, 124.4, 202.7, 116.6, 102.1 and 100%).

Regarding cell viability (green color) in brain regions of the limbic system, such as PFC, CA1, CA3, DG, ABL, and NAcc in animals fed with HCD, HFD, and their combination, as well as with CD, the results are shown in Fig. 8C. Qualitative analysis showed that in the three groups of foods high in carbohydrates, fats, and their combination, the red color was more intense in all brain regions studied, while the group fed with CD showed greater intensity of green and fewer red spots in the brain regions of the limbic system. The semi-quantitative analysis of Fig. 8D shows that the % of cell viability (green color) decreased in the PFC (*p* < 0.001; F = 124.6), CA1 (*p* < 0.001; F = 119.2), CA3 (*p* < 0.001; F = 97.38), DG (*p* < 0.001; F = 138.8), BLA (*p* < 0.001; F = 85.02) and NAcc (*p* < 0.001; F = 23.31) in the group with HCD (22.9; 20.6; 22.9; 25.8; 7.7 and 27.6%), HFD (16.9; 12.3; 20.2; 22.9; 5; 19.7%) and HCD + HFD (32.9; 22.1; 24.3; 31.2; 15.7; 31.8%,). In an integrated analysis, we can suggest that the consumption of diets high in carbohydrates, fats, or their combination, could represent a factor in increasing cell death by apoptosis.

## Discussion

The present study demonstrates that metabolic syndrome (MS) induced by chronic consumption of foods rich in carbohydrates, fats and their combinations exacerbate anxiety-like behaviors and decreases dendritic morphology in regions of the limbic system of male rats. This indicates that MS contributes to the negative alteration of neuronal limbic function, which is fundamental for the induction of anxious behaviors in male rats.

MS is characterized by a set of phenotypic alterations that reflect changes in body composition, including increased weight and fat mass (Neeland et al. 2024, Saklayen 2018). Alterations in carbohydrate, lipid, and hormonal profiles are also observed. The first objective of our work was to demonstrate that 12 weeks of high-fat and high-sugar consumption replicated the characteristics of MS in our biomodels at the zoometric, biochemical, and endocrine levels. The combination of a high-carbohydrate and a high-fat diet resulted in the highest fat mass percentage compared with control subjects, according to research (Bernard and Spalding 2023).

The body stores energy through adipocyte hypertrophy and hyperplasia when metabolic pathways are altered by excess energy intake (Choe et al. 2016). Fat storage can occur through two mechanisms: adipocyte hypertrophy, which increases cell size, and hyperplasia, which generates new adipocytes from precursor cells (Choe et al. 2016, Rosen and Spiegelman 2014). All of this causes cellular damage and impaired metabolic function, leading to lipotoxicity and insulin resistance (Yazıcı et al. 2024).

Insulin resistance is a hallmark of metabolic diseases. It is a complex disorder with broad etiological roots and diverse biochemical, molecular, and physiological manifestations (Saltiel and Kahn 2001, Shulman 2000). It is characterized by decreased insulin action, reduced glucose uptake, and compensatory hyperinsulinemia that can deplete pancreatic reserves. This impairment also compromises the inhibition of gluconeogenesis, promoting excessive peripheral glucose utilization (Esser et al. 2020, Hudish et al. 2019).

The biomodels treated with HCD, HFD, and their combination show clear signs of insulin resistance. The condition leads to elevated blood glucose, insulin, and fructosamine levels, which serve as indicators of hyperglycemia (Oliveira Andrade et al. 2023, Tomaszewska et al. 2024). Additionally, decreased insulin sensitivity and increased HOMA-IR are observed. These findings indicate that HCD, HFD, and HCD + HFD impair the ability to alternate energy substrates in cellular metabolism under energy-deficient conditions. Therefore, sustained pancreatic stimulation for glycemic control in animals is unsuccessful. Insulin resistance, in particular, is more evident in the HCD and HCD + HFD groups.

The lipid profile of animals that consume HCD, HFD, and their mixture shows clear signs of dyslipidemia because their bodies produce more endogenous lipids through *de novo* synthesis when they consume excessive amounts of glucose or lipids. The observed metabolic dysfunction proves that the body has entered a state of metabolic disorder (Vollenweider et al. 2015, Softic et al. 2016, Smith et al. 2020). The metabolic condition worsens when people consume diets high in carbohydrates. Our research shows that male rats consuming diets rich in carbohydrates, fats, and their combination develop MS symptoms that match the biochemical and zoometric characteristics of this disorder (see Fig. 1) .

After characterizing MS in our biomodels, the next objective of the study was to elucidate the controversy regarding the relationship between MS and hypothalamic-pituitary-adrenal (HPA) axis dysfunction (Pasquali et al. 2006). Our findings, interestingly, demonstrate that MS induced by HCD, HFD, or HCD + HFD exacerbates HPA axis activation, representing a sustained metabolic alteration that triggers considerable adrenal physiological stress. Reports indicate that corticosterone is the primary glucocorticoid in rats and is released in response to chronic stress (Joëls et al. 2018). This work shows that serum levels of corticosterone and ACTH, are significantly elevated, suggesting physiological adrenal stress. Several mechanisms could explain how MS leads to HPA axis overactivation in our animals. It is proposed that chronic low-intensity inflammation (metainflammation) and oxidative stress arising from dysfunctional adipose tissue are responsible for this physiological event (Bauer and Teixeira 2019). It has been reported that cytokines and inflammatory mediators from visceral and perirenal adipose tissue generated under MS conditions can reach neighboring tissues, such as the adrenal glands, through paracrine signaling, altering adrenal homeostasis (Infante et al. 2017, Zoulikha et al. 2021). Likewise, in our work group, it has been demonstrated that under MS conditions, neuroinflammation occurs in different brain regions (Treviño et al. 2022). In particular, hypothalamic inflammation enhances corticotropin-releasing hormone (CRH) synthesis and, in turn, alters the HPA axis (Herman et al. 2016). Likewise, metainflammation in the limbic system causes damage to neuronal structure and function, which, in the long term, would impact emotional behavior in both rodents and humans (Komleva et al. 2020).

HPA axis overactivation has been linked to heightened anxiety states (Herman et al. 2016, Flandreau et al. 2012). This work provides promising results regarding the relationship between MS and HPA axis activation, as well as its link to anxiety. Behavioral tests confirmed that animals with MS (consumption of diets rich in carbohydrates, lipids, and their mixture) developed anxiety-like behaviors, both under stressful and non-stressful conditions. The forced swim test under stressful conditions clearly shows that animals with high nutrient intake exhibit more anxiety-like behaviors. This behavior animals could be explained by the fact that chronic consumption of HFD and HCD increases mesolimbic dopaminergic activity and stress reactivity (Kleinridders and Pothos 2019, Wallace et al. 2021). Excessive food consumption can lead to weight gain and anxiety. In this context, we propose that MS could contribute as an additional factor that increases HPA axis activity and generates physiological stress in male rats. This stress compromises brain homeostasis and causes alterations in the structure and function of the limbic system, particularly in regions such as the prefrontal cortex, hippocampus, basolateral amygdala, and nucleus accumbens. It is noteworthy that these alterations could trigger behavioral changes in animals, such as compulsive eating and emotional dysregulation, this was evidenced by the increase in anxious behavior (Gomes et al. 2020, Dutheil et al. 2016).

Furthermore, the literature indicates that chronic overactivation of the HPA axis causes reduced neuronal plasticity (Herman et al. 2016). In this regard, we implemented Golgi-Cox staining in the brains of our MS biomodels. Sholl analysis demonstrated reduced dendritic complexity in pyramidal neurons of the prefrontal cortex, hippocampus, and basolateral amygdala, and in medium spiny neurons of the nucleus accumbens in MS animals. Something similar to reports indicating that exposure to saturated fats decreases dendritic morphology in layer 5 pyramidal neurons of the prefrontal cortex and in hippocampal neurons (Wainwright et al. 1998, Mota et al. 2023, Maniam et al. 2016). However, these results have the limitation that they do not replicate the biochemical and zoometric conditions of MS.

For this reason, it is essential to note that the HPA axis remains activated all the time, as eating foods high in fat and carbohydrates, or their mix, leads to ongoing inflammation and metabolic problems. Prolonged exposure to corticosterone leads to decreased neurotrophic factor production, which eventually results in damage to limbic system neuronal dendrites. These brain regions exhibit changes in their neural pathways due to structural modifications (Hassamal 2023, Boitard et al. 2015). The environment becomes more suitable for anxious behavior development because dysfunctional emotional regulation exists. The basolateral amygdala operates independently because it directly regulates anxious behaviors. The brain region becomes more prone to inflammation and altered excitability, disrupting neuronal plasticity and worsening anxious behaviors (Herman et al. 2016, Moreno-Martínez et al. 2022, Iemolo et al. 2013). Within the framework of this project, the basolateral amygdala stands out as one of the most affected regions, suggesting that the deterioration it presents may be contributing to the development of anxiety-like behaviors. Also, our research group’s results reveal that exposure to a 3-month carbohydrate-rich diet emulates MS conditions and reduces dendritic complexity in the hippocampus, prefrontal cortex, basolateral amygdala, and medium spiny neurons of the nucleus accumbens (Fuentes et al. 2023).

Some studies suggest that chronic intake of carbohydrate- and fat-rich diets activates the p75NTR receptor pathway in the brain, which is often associated with neuronal injury (Mohamed and El-Remessy 2015, Shen et al. 2014). This pathway promotes the release of proneurotrophins, which activate sortilin receptors, induce apoptosis, and interrupt retrograde signaling (Skeldal et al. 2012, Campagnolo et al. 2014). In addition to our results, we demonstrated that apoptosis and loss of viability occur in the limbic regions studied in animals with MS. This was evidenced by increased caspase-3 immunoreactivity, a key mediator of programmed cell death, and decreased cell viability, as assessed by acridine orange (Bressenot et al. 2009).

Currently, there is uncertainty about whether the results observed in male rats can be extrapolated to female rats. Research findings show that males and females exhibit distinct responses to high-fat and high-carbohydrate diets (Paula et al. 2024, Myles et al. 2023, Abi-Ghanem et al. 2023). The scientific literature shows that males develop more visceral fat, along with insulin resistance and metabolic issues, whereas females exhibit different body measurements and changes in blood chemistry (Noronha et al. 2019, Maric et al. 2022). The research shows that sex differences exist in how brain inflammation and neuronal damage affect the development of anxiety symptoms (Noronha et al. 2019, Muscat et al. 2023). The neuroprotective effects of estrogen are crucial in female rats, as they influence inflammation, synaptic plasticity, and oxidative stress (Maioli et al. 2021). The presence of estrogen seems to protect neurons from damage, which might extend the time before anxiety symptoms appear and memory decline starts (Noronha et al. 2019, Boukouvalas et al. 2010). The current study acknowledges its limited scope, as it included only male subjects.

Finally, it is essential to note that the findings of this study could also occur in humans. The mechanisms of brain damage, such as neuroinflammation and HPA dysfunction observed in our biomodels, allow for a biologically plausible extrapolation to humans, both male and female (Saltiel and Olefsky 2017, McEwen and Gianaros 2010). Some neuroimaging studies show alterations in the human hippocampus associated with obesity or MS and show a relationship with anxiety symptoms (Holzschneider and Mulert 2011). However, the magnitude and characteristics of neuronal damage must be considered, as they are likely to differ by sex, age, and hormonal status. Hence, the importance of performing sex-stratified analyses in humans (Huang et al. 2020, Chella Krishnan et al. 2018, Dreux et al. 2025).

In conclusion, our results demonstrate for the first time that MS caused by chronic exposure to diets rich in carbohydrates, fats, and a combination of both in male rats constitutes a critical factor contributing to HPA axis dysfunction and dendritic hypotrophy in brain regions of the limbic system, which could impact the emergence of anxious behaviors. This also leads to impaired recognition memory (as reported previously by our research group. These findings support the idea that MS poses a significant risk to mental health and reduces quality of life. Therefore, MS is considered one of multiple factors that could contribute to the development of anxious behaviors and long-term neurological deterioration. It is important to note that further studies are needed, particularity those that include women, and that these findings should be extrapolated to people of different ages and sexes.

## Data Availability

The data that support the findings of this study are available from the corresponding author upon reasonable request.
